# Investigation of muscle transcriptomes using gradient boosting machine learning identifies molecular predictors of feed efficiency in growing pigs

**DOI:** 10.1186/s12864-019-6010-9

**Published:** 2019-08-17

**Authors:** Farouk Messad, Isabelle Louveau, Basile Koffi, Hélène Gilbert, Florence Gondret

**Affiliations:** 1 Pegase, INRA, Agrocampus Ouest, 35590 Saint-Gilles, France; 2 GenPhySE, INRA, 31326 Castanet Tolosan, France

**Keywords:** Feed efficiency, Machine learning, Muscle, Pig, Prediction, Transcriptome

## Abstract

**Background:**

Improving feed efficiency (FE) is a major challenge in pig production. This complex trait is characterized by a high variability. Therefore, the identification of predictors of FE may be a relevant strategy to reduce phenotyping efforts in breeding and selection programs. The aim of this study was to investigate the suitability of expressed muscle genes in prediction of FE traits in growing pigs. The approach considered different transcriptomics experiments to cover a large range of FE values and identify reliable predictors.

**Results:**

Microarrays data were obtained from *longissimus* muscles of two lines divergently selected for residual feed intake (RFI). Pigs (*n* = 71) from three experiments belonged to generations 6 to 8 of selection, were fed either a diet with a standard composition or a diet rich in fiber and lipids, received feed ad libitum or at restricted level, and weighed between 80 and 115 kg at slaughter. For each pig, breeding value for RFI was estimated (RFI-BV), and feed conversion ratio (FCR) and energy-based feed conversion ratio (FCRe) were calculated during the test periods. Gradient boosting algorithms were used on the merged muscle transcriptomes to identify very important predictors of FE traits. About 20,405 annotated molecular probes were commonly expressed in *longissimus* muscle across experiments. Six to 267 expressed muscle genes covering a variety of biological processes were found as important predictors for RFI-BV (*R*^2^ = 0.63–0.65), FCR (*R*^2^ = 0.61–0.70) and FCRe (*R*^2^ = 0.49–0.52). The error of prediction was less than 8% for FCR. Altogether, 56 predictors were common to RFI-BV and FCR. Expression levels of 24 target genes were further measured by qPCR. Linear regression confirmed the good accuracy of combining mRNA levels of these genes to fit FE traits (RFI-BV: *R*^2^ = 0.73, FRC: *R*^2^ = 0.76; FCRe: *R*^2^ = 0.75). Stepwise regression procedure highlighted 10 genes (FKBP5, MUM1, AKAP12, FYN, TMED3, PHKB, TGF, SOCS6, ILR4, and FRAS1) in a linear combination predicting FCR and FCRe. In addition, FKBP5 and expression levels of five other genes (IGF2, SERINC3, CSRNP3, EZR and RPL16) significantly contributed to RFI-BV.

**Conclusion:**

It was possible to identify few genes expressed in muscle that might be reliable predictors of feed efficiency.

**Electronic supplementary material:**

The online version of this article (10.1186/s12864-019-6010-9) contains supplementary material, which is available to authorized users.

## Introduction

Improving feed efficiency (FE) is an utmost challenge for the profitability of pig production with additional benefits on its ecological footprint. In production farms, FE during growth is assessed by its inverse trait, the feed conversion ratio (FCR) calculated as daily feed intake divided by daily growth rate over a defined period. Residual feed intake (RFI) has been specifically proposed to capture the efficiency of feed use independent from the production needs [1], corresponding to the so-called net feed efficiency. The RFI can be computed at the genetic or phenotypic levels as the difference between observed feed intake and feed intake predicted from production and maintenance needs. Thanks to a moderate (~ 0.40) genetic correlation between RFI and FCR, selection experiments for RFI have been successful in generating low and high RFI divergent lines, which also displayed a large difference in FCR [2, 3]. Several high-throughput studies based on microarrays [4–8] and RNA sequencing [9–12] have been conducted on small numbers of pigs with extremely low or high RFI phenotypes. These studies were helpful to ascribe how molecular pathways within and across tissues, namely muscle, adipose tissues, liver and intestine, can be related to variations in FE traits. Pathways related to oxidative stress response, inflammation, and immune system have been reported as consistently involved in the differences between RFI genetic lines [4] or extreme RFI phenotypes [11]. Identifying molecular hubs in co-expression networks of genes associated with high and low FE traits has been also considered to propose candidate biomarkers in the liver or duodenum in pigs and cows [11, 13]. Finally, machine learning algorithms have gained increasing attention to handle high dimensional datasets where the number of potential explanatory variables vastly exceeds the number of observations, and to select an optimal subset of variables for classification or prediction of particular phenotypes. The importance scores generated by random forest and support vector machines, two methods considered as state-of-the art of machine learning algorithms, have been recently applied to whole-genome molecular markers for prediction of RFI in beef cattle [14, 15]. Support vector machine, random forest, elastic net, and nearest shrunken centroid algorithms have been also successfully tested for their ability to classify extreme pigs on high/low RFI from RNAseq data in liver and duodenum [16]. When applied on microarray datasets from human subjects to predict health outcomes [17], the best prediction accuracy was obtained for the gradient tree boosting machine (GTB) among seven machine learning approaches including random forest and support vector machine. This algorithm is considered to produce an excellent fit of predicted to observed values, even if the specific nature of the relationships between the predictor variables and the dependent variable is very complex. This suggests that this approach can be suitable for regression problems such as the prediction of FE values.

The numbers of differentially expressed genes between low and high RFI pigs were found much higher in muscle than in the liver or adipose tissues [4], suggesting that muscle may be a relevant target to unravel the complexity of FE. However, very few overlaps were found between lists of differentially expressed muscle genes from different studies [8, 9]. The amount and type of feed offered, energy supply, animal sex and body weight, season of rearing, etc. interact with the genetic background to influence variations in RFI and other FE traits [18]. Likely, this adds to the difficulty to find common candidates to explain and predict FE. Combining different molecular datasets to provide a larger number of animals and wider ranges of experimental conditions may be a relevant strategy to obtain a robust description of predictors involved in the phenotypes of interest [19, 20]. This study aimed to identify important muscle genes for prediction of FE in growing pigs. Experiments run on two pig lines divergently selected for RFI that have been already analyzed separately [4, 8] and included differences in selection generations, feed allowance and diet composition, sex and live weight of the pigs were combined. The GTB algorithms were then used as a resampling machine learning approach to re-examine muscle microarray datasets and to predict different FE traits.

## Results

### Descriptive statistics on merged molecular datasets

**Fig. 1a:**
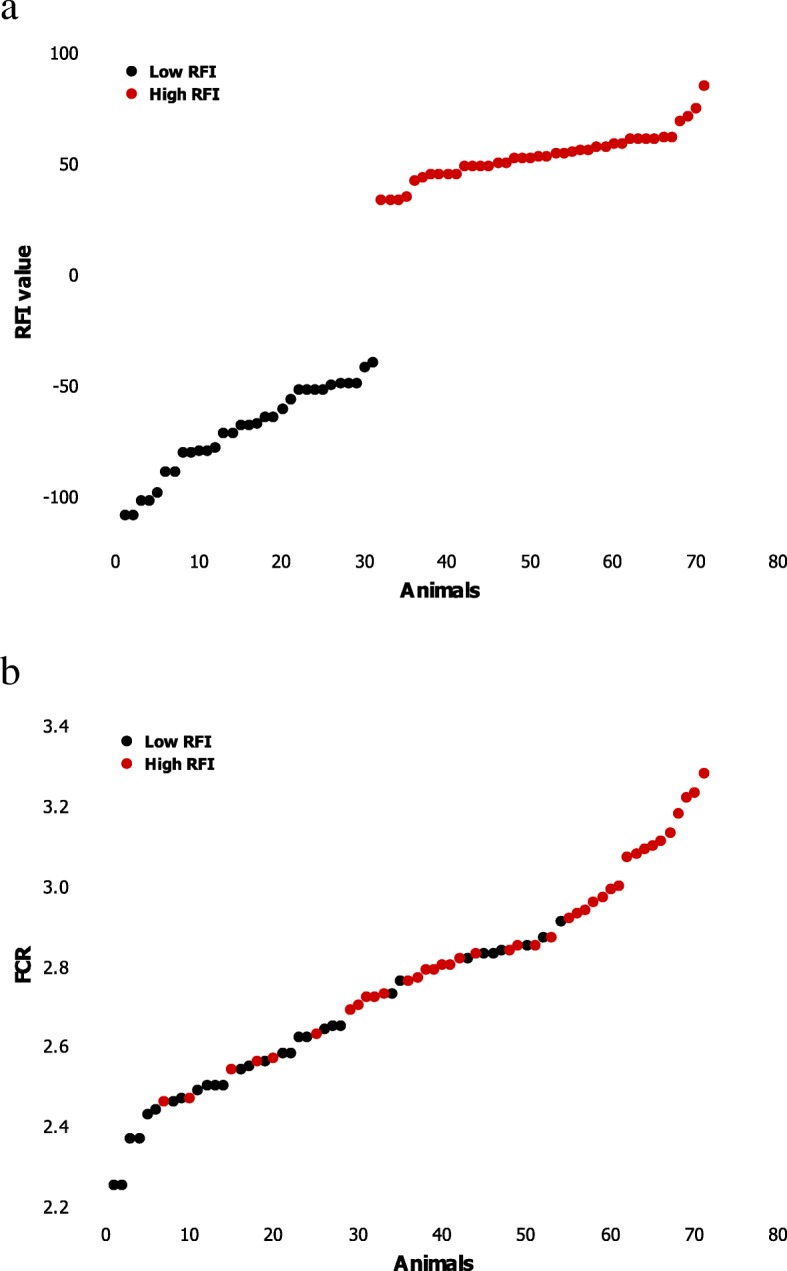
Distribution of residual feed intake (RFI) **b** Distribution of feed conversion ratio (FCR). Barrows and females growing pigs from generations 6 to 8 of a divergent selection for RFI were considered. Pigs from the low or high RFI lines were fed different diets according to referenced publications [21, 22]. Black dot blot: pigs of the low RFI line; red dot blot: pigs of the high RFI line

Microarrays data from *longissimus* muscle of 71 barrows and female pigs of two lines divergently selected for RFI and reared under different experimental conditions were reanalysed from available molecular repositories. A total of 20,405 expressed annotated molecular probes were successfully matched over repositories and included in a new merged dataset. In this new dataset, pigs were ascribed to low or high RFI groups according to their genetic lines. Traits related to FE were available from references publications [21, 22] or newly calculated from data obtained on littermates in the selection farms [3]. Descriptive statistics are shown in Table 1. The RFI breeding values (RFI-BV) ranged from − 108.7 to 91.6 g/d, and the mean value was significantly lower, as expected from selection, for pigs of the low RFI line than for pigs of the high RFI line. The FCR was 2.75 kg/kg on average (min = 2.25; max = 3.28). It was 27 MJ/kg BW on the net energy basis (FCRe: min = 22; max = 32). As expected, pigs of the low RFI line had lower feed conversion ratios (FCR, FCRe) than pigs of the high RFI line (*P* < 0.001). Because pigs were reared under different experimental conditions, a large range of values for feed intake (FI) was observed (min =1725 g/day; max = 3026 g/day). A large range of FCR values was covered in the merged dataset, with some interpenetration between pigs of the low and high RFI lines (Fig. 1).

**Table 1:** Descriptive statistics for feed efficiency traits and growth performance

Variable	Line	*n*	Mean	SEM	StDev	Minimum	Maximum
RFI-BV	Low RFI	31	− 66.5^a^	3.6	20.1	−108.7	−39.5
	High RFI	40	55.9^b^	1.7	10.9	33.5	91.6
ADG	Low RFI	31	885.0^a^	15.9	88.7	700	1068
	High RFI	40	827.4^b^	15.7	99.2	543	1012
FI	Low RFI	31	2288.3^a^	34.2	190.4	1914	2658
	High RFI	40	2362.7^a^	40.5	255.9	1725	3026
FCR	Low RFI	31	2.60^b^	0.03	0.18	2.25	2.91
	High RFI	40	2.87^a^	0.03	0.21	2.46	3.28
FCRe	Low RFI	31	25^b^	0.3	2	22	29
	High RFI	40	28^a^	0.3	2	24	32

*Abbreviations used*: *ADG* Average daily gain (g/d), *FI* feed intake (g/kg), *FCR* Feed conversion ratio (kg/kg), *FCRe* Net energy feed conversion ratio (MJ/kg), *RFI-BV* Breeding value for residual feed intake (g/d). Data were obtained in *n* = 71 growing pigs from two lines divergently selected for residual feed intake (low/high) and reared under different conditions. ADG, FI and FCR were obtained from referenced publications [21, 22]. The FCRe was newly calculated using the net energy content of diets that was provided in the same publications. Genetic RFI values were newly calculated from performance recorded on pig littermates reared in the selection farm (Rouillé, France). For each trait, data obtained from pigs of both lines were compared by ANOVA; a, b: for a given trait, means with different superscript letter differed between low and high RFI lines (*P* < 0.05)

**Fig. 2:**
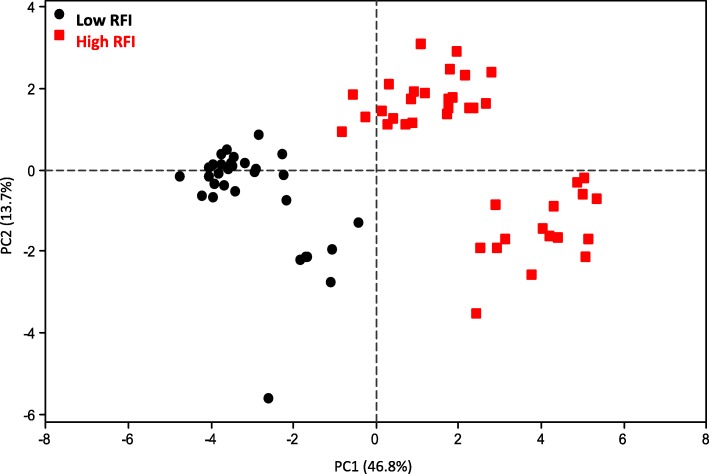
Plot of the first two principal components unraveling whole variability in the merged molecular dataset. Pigs are represented on the scatter plot created with the first two principal components (PC) of a Principal Component analysis (PCA) which aggregated the whole transcriptomic data (20,405 annotated expressed probes) in the *longissimus* muscle of different studies. The first PC of the PCA (PC1) represented 46.8% of the whole transcriptomic variability and discriminated pigs from the low or high RFI selection lines. This allows considering PC1 of the PCA as a relevant summary of the main molecular probes involved across the pigs in divergence for RFI. Black dot blot: pigs of the low RFI line; red dot blot: pigs of the high RFI line black dot blot: pigs of the low RFI line; red dot blot: pigs of the high RFI line

Principal Component Analysis (PCA) was then used to handle the 20,405 expressed probes in *longissimus* muscle on a common frame, to detect outliers and visualize links between variables. The first principal component (PC1) summarized 46.8% of the observed variance, whereas the second principal component (PC2) summarized 13.7% of the variability. Pigs of the low RFI group were opposed to pigs of the high RFI group on PC1 (Fig. 2), whereas PC2 did not allow any distinction between the two RFI groups. The FCR was significantly (*P* < 0.001) correlated with PC1 (*r* = 0.53) and PC2 (*r* = − 0.41). The FCRe was also significantly (*P* < 0.001) correlated with PC1 (*r* = 0.49) and PC2 (*r* = − 0.44). The partition of pigs due to their genetic lines (low or high RFI) and the datasets of origin is shown in Suppl. File 1.

### Selection of very important expressed muscle genes to predict feed efficiency

Gradient Tree Boosting (GTB) was applied as a machine learning method that processes with thousands decision trees to successively produce prediction models for RFI-BV, FCR and FCRe with relevant subsets of annotated probes called very important variables in prediction (VIP) for these traits. Conditional accuracy parameters (RMSE, R^2^) were estimated for each FE trait (Table 2). The lists of identified VIP and their corresponding scores in prediction models were provided into Additional file 2 for RFI-BV, Additional file 3 for FCR and Additional file 4 for FCRe, respectively.

**Table 2:** Number of probes and encoded genes identified as VIP for feed efficiency traits

	Nb annotated probes	Nb unique genes	R^2^	RMSE
RFI-BV	384	222	0.63	42.9
	280	161	0.64	39.6
	50	27	0.65	39.3
FCR	421	267	0.61	0.23
	88	52	0.70	0.22
	50	33	0.67	0.22
FCRe	318	218	0.49	2.2
	50	29	0.52	2.0
	7	6	0.52	2.0

Machine learning procedure (gradient tree net boosting) was applied on microarrays dataset (20,405 expressed annotated probes) generated from the *longissimus* muscle of 71 growing pigs to identify models able to predict residual feed intake (RFI), feed conversion ratio (FCR) and net energy-based feed conversion ratio (FCRe). A randomly selected bootstrap pig sample (*n* = 50) was used for learning, whereas the remaining pigs (*n* = 21) was used for validation test. The first rounds led to model stabilization with 384 molecular probes as very important variables (VIP) for RFI-BV prediction, 421 probes for FCR prediction and 318 probes for FCRe prediction, respectively, out of the 20,405 expressed annotated probes. The second entry was an iterative step of the former procedure but considering the VIP that were identified in the first step as the new inputs. This increased the accuracy of the prediction evaluated by the root mean square error (RMSE) and the coefficient of determination (R^2^). The last entry was another iterative step using the VIP identified at the second step as the new inputs, which led to identify the smallest number of VIP able to predict the target trait with a good accuracy. The numbers (Nb) of annotated probes and their corresponding unique genes identified as VIP for the three feed efficiency traits were indicated. Lists of the VIP (probes and their corresponding gene name when applicable) are provided in Additional files 1, 2 and 3

TreeNet boosting procedure was applied to 20,405 annotated probes expressed in the longissimus muscle of 71 pigs to release very important predictors (VIP) that can be used to predict residual feed intake (RFI) values. A total of 384 molecular probes were identified. Iterative steps led to reduce the set to 50 molecular probes corresponding to 30 unique encoded genes. These genes were listed by the order of importance (score) in prediction. Expression levels of genes indicated in bold face were further measured by qPCR

For RFI-BV, 384 molecular probes corresponding to 222 unique annotated genes were first identified as VIP in the predictive model (*R*^2^ = 0.63). Biological meaning of these VIP was further explored using DAVID bioinformatics resources. Fifteen biological pathways (cutoffs: enrichment score E > 1, *P* < 0.05) were listed (Table 3). They corresponded to enzyme linked receptor protein signaling pathway, regulation of cell motion and cell growth, responses to hormone stimulus, inorganic substance or nutrient levels, the regulation of homeostatic process and different metabolic related processes (hexose metabolic process, glycerol-phospholipid metabolic process, organic acid biosynthetic process and protein amino acid phosphorylation), regulation of muscle development, and the inflammatory response. Iterative steps allowed to increase the accuracy of the model with a reduced number of variables, so that 50 molecular probes corresponding to 27 unique genes were further proposed as top VIP (*R*^2^ = 0.65).

**Table 3:** Main overrepresented biological processes shared by genes selected as predictors of feed efficiency traits

GO terms	Nb genes	E	*P* Value	Clustered genes
RFI (clustered pathways among 222 VIP)				
GO:0051270~regulation of cell motion	12	3.35	< 0.001	BCL2, F10, HBZGF, HDAC5, **INS-IGF2**, LAMA4, NTN1, NRP1, PIK3R1, PDGFRB, SERPINE2, TGFR3
GO:0007167~enzyme linked receptor protein signaling pathway	14	2.36	< 0.001	AMHR2, NRP1, **INS-IGF2**, TIPARP, TRIO, GRB10, UTP11L, PDGFRB, SPTBN1, HBEGF, ROR2, TGFBR3, ANGPTL1, PIK3R1
GO:0001558~regulation of cell growth	8	1.86	0.008	NRP1, CD44, **INS-IGF2**, ABTB2, BCL2, HBEGF, NTN1, MAP 2 K5
GO:0009725~response to hormone stimulus	12	1.81	0.004	HDAC5, PLA2G4A, AR, GRB10, CCND2, **INS-IGF2**, BCL2, NCOA6, TGFBR3, MGP, CA2, PIK3R1
GO:0019318~hexose metabolic process	8	1.79	0.008	PDK1, TPI1, **PHKB**, **INS-IGF2**, **UGDH**, FUT2, SLC35A2, PMM1
GO:0007517~muscle organ development	8	1.79	0.01	SRPK3, GATA6, TIPARP, PDGFRB, TGFBR3, HBEGF, ZFPM2, CBY1
GO:0032844~regulation of homeostatic process	5	1.38	0.05	PLA2G4A, CD44, BCL2, RYR2, CA2
GO:0060284~regulation of cell development	10	1.33	< 0.001	HDAC5, NRP1, **PSEN1**, CCND2, **INS-IGF2**, BCL2, HOXD3, RTN4R, TGFBR3, NTN1
GO:0048705~skeletal system morphogenesis	7	1.31	0.004	TULP3, **PSEN1**, HOXD3, TIPARP, PDGFRB, ROR2, MGP
GO:0006468~protein amino acid phosphorylation	15	1.30	0.03	SRPK3, PDK1, AMHR2, TWF1, TRIO, ADRBK1, CDKL2, **PSEN1**, BCL2, SPTBN1, ROR2, PDGFRB, TGFBR3, MERTK, MAP 2 K5
GO:0010035~response to inorganic substance	9	1.24	0.003	ACTB, PLA2G4A, SLC1A3, BCL2, UROS, RYR2, MGP, ADRBK1, CA2
GO:0006954~inflammatory response	8	1.20	0.08	HDAC5, CD44, **INS-IGF2**, FCN2, TICAM2, **PSEN1**, NLRP3, NFX1
GO:0031667~response to nutrient levels	8	1.10	0.08	PLA2G4A, **PSEN1**, CD44, BCL2, RYR2, MGP
GO:0006650~glycerophospholipid metabolic process	5	1.08	0.08	PLA2G4A, ABHD5, ADNP, LPCAT2, PIK3R1
GO:0016053~organic acid biosynthetic process	6	1.08	0.04	TPI1, SLC1A3, SCD, ABHD5, UROS, **UGDH**
FCR (clustered pathways among 267 VIP)				
GO:0007010~cytoskeleton organization	13	2.4	0.001	RND3, ACTC1, **EZR**, MACF1, CALD1, BCL2, SSH2, KRT8, ABI2, CNN1, TTN, PRKG1, EPB49
GO:0007155~cell adhesion	20	1.85	0.011	TECTA, NRP1, OLR1, GMDS, LGALS4, CNKSR3, NLGN3, CLDN10, CLDN11, CD84, RND3, LAMA4, **EZR**, ROBO1, COL27A1, BCL2, ACAN, MSN, **PDZD2**, EDA
GO:0060284~regulation of cell development	8	1.78	0.038	NRP1, LYN, ROBO1, **INS-IGF2**, BCL2, HOXD3, SMAD3, **IGF2**, NTN1
GO:0006006~glucose metabolic process	7	1.66	0.03	TPI1, PYGM, **PHKB**, PYGL, INS-IGF2, SDS, **UGDH**, **IGF2**
GO:0060537~muscle tissue development	8	1.66	0.003	MYF6, ACTC1, GATA6, TIPARP, TTN, CHRNA1, HOMER1, PTEN
GO:0005977~glycogen metabolic process	3	1.61	0.099	PYGM, **PHKB**, PYGL
GO:0000902~cell morphogenesis	10	1.50	0.095	**EZR**, NRP1, SEMA6C, MACF1, ROBO1, BCL2, LIFR, SOX6, NTN1, MYCBP2
GO:0001568~blood vessel development	9	1.42	0.034	CCM2, LAMA4, NRP1, ROBO1, TIPARP, TGFA, DBH, FIGF, PTEN
GO:0045321~leukocyte activation	8	1.35	0.077	LYN, **INS-IGF2**, **FYN**, BCL2, SMAD3, MALT1, **IGF2**, HSPD1, ZNF3
GO:0001501~skeletal system development	10	1.29	0.055	GNAQ, **INS-IGF2**, GFPT1, BCL2, HOXD3, TIPARP, ACAN, SMAD3, GNAS, **IGF2**, SOX6
GO:0016052~carbohydrate catabolic process	6	1.27	0.025	GPD1L, OVGP1, TPI1, PYGM, PYGL, FUT1
GO:0030163~protein catabolic process	15	1.19	0.093	FEM1C, SOCS3, WWP1, USP9X, **SOCS6**, HECTD2, MALT1, ASB13, SMURF1, UBE2J2, SPOPL, UBE2Q1, USP32, MYCBP2, RNF111
FCR (clustered pathways among 218 VIP)				
GO:0051270~regulation of cell motion	15	1.95	0.001	RET, MSH2, MDGA1, ARID5B, NR4A2, KDR, DSTN, IGSF8, MACF1, **FYN**, BAX, PAK4, FOXE1, THBS1, ACVR1
GO:0034613~cellular protein localization	13	1.58	0.004	COPA, CLTA, YWHAZ, LTBP2, AP1G1, **AKAP12**, PTPRU, SYNGR1, MACF1, RPL23, BAX, CHM, RAB11A
GO:0006163~purine nucleotide metabolic process	7	1.54	0.02	**ATP1B1**, ENPP1, MSH2, ATP1B4, RAB11A, ACLY, MYH7
GO:0001568~blood vessel development	9	1.49	0.009	EPAS1, BAX, CHM, ZFPM2, TNNI3, THBS1, MMP2, KDR, ACVR1
GO:0006732~coenzyme metabolic process	6	1.25	0.04	DLD, ACLY, ALDH1L2, GCLM, MTHFD1L, MOCS1
GO:0042592~homeostatic process	17	1.22	0.02	ENPP1, EPAS1, PTH1R, PRDX3, TNNI3, GCLM, MBP, KDR, RPS19, SLC4A11, RHCG, IL20RB, BAX, DLD, FABP4, IKBKB, CLN6
GO:0003006~reproductive developmental process	8	1.10	0.04	HSPA2, MSH2, BAX, DLD, SF1, DHCR24, KDR, ACVR1
GO:0048514~blood vessel morphogenesis	7	1.08	0.04	EPAS1, BAX, ZFPM2, TNNI3, THBS1, KDR, ACVR1
GO:0043066~negative regulation of apoptosis	11	1.04	0.01	YWHAZ, MSH2, BAX, BTC, NR4A2, PRDX3, IKBKB, THBS1, GCLM, DHCR24, ACVR1

Very important genes (VIP) for prediction of feed efficiency traits (RFI: residual feed intake; FCR: feed conversion ratio; FCRe: net energy based-feed conversion ratio). Genes were clustered into functional groups using DAVID tool. The enrichment score (E > 1) for each cluster and *P*-value of the enrichment for the corresponding Gene Ontology (GO) terms are provided. Expression levels of genes indicated in bold font were further measured by qPCR

For FCR, 421 molecular probes corresponding to 267 unique identified genes were identified as VIP (*R*^2^ = 0.61; RMSE = 0.23). Twelve biological pathways were listed across VIP (Table 3). They were related to energy metabolic process (glucose metabolism, glycogen metabolic process, carbohydrate catabolic process) and protein catabolic process, regulation of muscle development (muscle tissue development, skeletal tissue development, regulation of cell development, cell morphogenesis), cell adhesion and cytoskeleton organisation, blood vessel development, and leukocyte activation. Model reduction further led to identify a subset of 50 probes corresponding to 33 unique genes with an increased accuracy of the prediction (*R*^2^ = 0.67; RMSE = 0.22). Thus, the error of prediction was 8% of the mean of the trait.

For FCRe, 318 probes corresponding to 218 unique genes were retained in the prediction model (*R*^2^ = 0.49; RMSE = 2.0). Nine biological pathways (enrichment score E > 1, *P* < 0.05) were listed across VIP for FCRe (Table 5). They were homeostatic process, coenzyme metabolic process, purine nucleotide metabolic process, cellular protein localization, regulation of cell motion, blood vessel development and blood vessel morphogenesis, reproductive developmental process, and negative regulation of apoptosis. Iterative steps led to a drastic reduction of the predictors with only 7 probes corresponding to 6 unique identified genes identified as top VIP (*R*^2^ = 0.52; RMSE = 1.9). The error of prediction was 7% of the mean of the trait.

The GTB algorithm was also applied to predict ADG, but model performance was lower (*R*^2^ = 0.45 and RMSE = 70.32; data not shown) than for feed efficiency traits. This means that the prediction was better for composite traits than for individual traits in this situation.

### Common VIP to predict RFI, FCR and FCRe

**Fig. 3:**
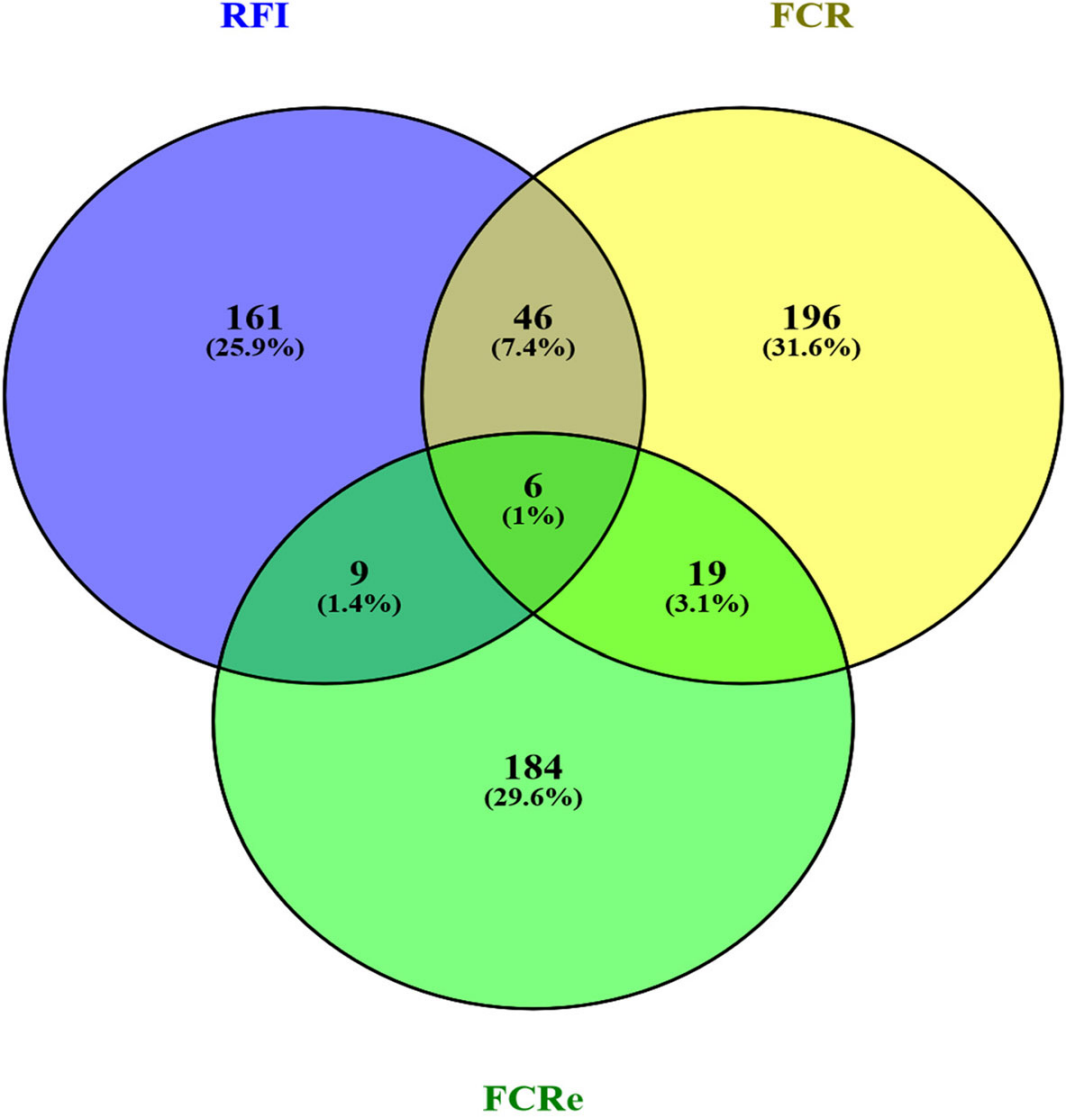
Venn diagrams to identify commonalities between lists of VIP for feed efficiency trait. Predictive models were built from microarrays transcriptomics dataset to identify the most important annotated expressing probes in the *longissimus* muscle able to predict breeding values of RFI, and feed-conversion-ratio (FCR) and net energy-based feed conversion ratio (FCRe) values. The lists of these probes identified as VIP (very important variables in prediction) were then uploaded by their corresponding gene name in the VENNY tool. Venn diagram was edited to enlighten commonalities between the lists of unique genes identified as VIP for the three traits

Lists of VIP proposed in the models for the different FE traits were handled into a Venn diagram (Fig. 3). There were 56 VIP in common for prediction of RFI and FCR, 15 common VIP between RFI and FCRe predictors, and 25 common VIP for FCR and FCRe. Finally, six VIP were found in common from the models predicting RFI, FCR and FCRe. The corresponding genes were listed in Table 4. When the smallest subsets of VIP obtained after models reduction were considered, none genes were common across the three traits.

**Table 4:** Lists of muscle genes identified as common predictors for feed efficiency traits

Traits	Common VIP
RFI/FCR	ANKRD1; ANKRD42; ARF3; BCL2; **BLCAP**; BNC2; C15orf40; CNN1; CREBRF; **CSRNP3**; DMTF1; EPHX1; **EZR;** FAM43B; **FKBP5**; **FRAS1**; GATA6; GPR153; HOXD3; **IL4R; INS-IGF2**; LAMA4; MACF1; MBTPS1; MX2; NRP1; NTN1; **PDZD2**; PFDN4; **PHKB**; POR; PSTK; QRSL1; RASL11B; RBP1; RPGR; SAMD4A; SDR39U1; SEPN1; **SERINC3**; SLC41A1; **SOCS6**; SYNE2; TICAM2; TIPARP; TPI1; TRIM38; **UGDH**; UROS; ZNF280D; ZNF443; ZNF644
RFI/FCRe	**AKAP12**; ANKRD1; DMTF1; **EZR**; FCN2; **FKBP5**; MACF1; NCOA6; NGB; OAZ3; SLC35A2; TNFRSF21; ZFAND3; ZFPM2; ZNF644
FCR/FCRe	ANKRD1; **ATP1B1**; BVES; C4orf21; CCDC91; DMTF1; **EZR; FKBP5**; FOXN3; **FYN;** GMDS; GZMK; HIST1H2BD; HSPA2; KCNJ2; LOC100505669; MACF1; MEF2A; MTHFD1L; RAB28; **RPL6;** SEMA4A; **SERPINA1**; WWP1; ZNF644
RFI/FCR/FCRe	ANKRD1; DMTF1; **EZR; FKBP5**; MACF1; ZNF644

Very important expressed muscle genes (VIP) identified as important for prediction of residual feed intake (*n* = 384 VIP), feed conversion ratio (FCR, *n* = 421 VIP) or net energy based-feed conversion ratio (FCRe, *n* = 318 VIP) were indicated. Genes indicated in bold font were further considered for qPCR analysis

### Model evaluation for FCR prediction

**Fig. 4:**
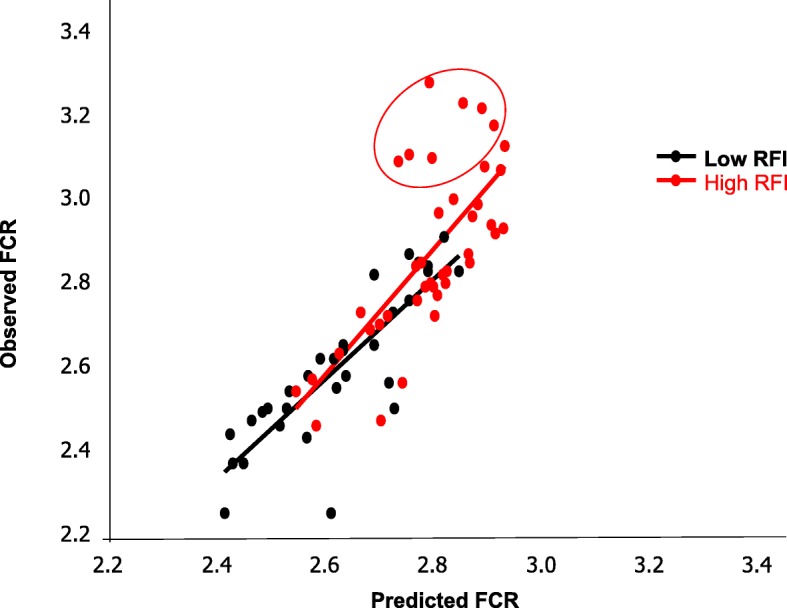
Regression between observed and predicted FCR values. The predictive model was built from microarrays transcriptomics dataset by TreeNet boosting procedure to identify the most important annotated expressing probes in the *longissimus* muscle able to predict feed-conversion-ratio (FCR) from *n* = 71 pigs of two divergent selection lines for residual feed intake (RFI). The graph was then computed between observed and predicted FCR values. The red line represents pigs of the high RFI group, the black line represents pigs of the low RFI group. Bad prediction concerned seven pigs (encircled) of the high RFI line

Because FCR is the most widely used indicator of FE at the farm level, a deeper evaluation of the model performance was carried out for this trait. Predicted (X) and observed (Y) values were compared (X - Y), using the GLM procedure. The model was considered unbiased when the intercept was not different from 0 and the slope was not significantly different from 1, and the quality of the relationships was also evaluated on the basis of RMSE of prediction (RMSEP) obtained by a leave-one-out cross-validation from the value of the predicted residual sum of squares. Observed and predicted values for FCR were close together when evaluated on all pigs (*R*^2^ = 0.70, RMSEP = 0.13; Fig. 4). When examined by RFI line, intercept and slope of the regression line were not significantly different from 0 and 1, respectively, for pigs of the low RFI line (*R*^2^ = 0.71; RMSEP = 0.09), but the intercept was significantly (*P* < 0.05) different from 0 for pigs of the high RFI line (*R*^2^ = 0.51; RMSEP = 0.15). Bad prediction concerned 7 pigs (*R*^2^ = 0.27), which belonged to the high RFI line and had very high FCR values (3.17 kg/kg on average). They originated from different experiments including 2 pigs feed-restricted during the growing-finishing period and 5 pigs fed ad libitum either a control diet or a high-fat high-fiber diet. When these 7 pigs were removed, the quality of the prediction for the remaining animals (*n* = 64) was clearly improved (*R*^2^ = 0.80; RMSEP = 0.09), and no more difference was observed in model prediction quality between low RFI (*R*^2^ = 0.71; RMSEP = 0.10) and high RFI (*R*^2^ = 0.75; RMSEP = 0.09) lines. A detailed representation of the regression slopes when calculated within the RFI groups and datasets of origin is available in Additional file 5.

### Expression levels of target genes and linear regression models on feed efficiency traits

The mRNA levels of genes, identified as VIP for at least two FE traits or participating in pathways judged by expertise as biologically relevant in FE, were further measured by qPCR to provide further validation and to propose simplified regression models. Twenty-four target genes were chosen among the VIP, and the majority (75%) but not all of these genes had a differential expression (*P* < 0.05) as evaluated by variance analysis, between pigs of the low or high RFI groups (Table 5). Linear regression models with RSQUARE selection (independent variables that best predict the dependent variable by linear regression) or stepwise option (variables are added one by one to the model), were then applied. Linear combination of mRNA levels of the 24 target genes allowed to predict FE traits with very good accuracy (RFI-BV: *R*^2^ = 0.73, FRC: *R*^2^ = 0.76; FCRe: *R*^2^ = 0.75). Complementary analyses using final BW of pigs as an additional explicative variable in regression did not change the accuracy of prediction for feed efficiency traits (RFI-BV: *R*^2^ = 0.75, FRC: *R*^2^ = 0.77; FCRe: *R*^2^ = 0.76; data not shown). Finally, stepwise linear regression highlights a combination of mRNA levels of 10 or 11 genes to explain about 71% of the total variability in FCR and FCRe, respectively, whereas expression levels of 6 genes contributed to 58% of the variability in RFI-BV (Table 6).

**Table 5:** Average expression levels of target genes studied by qPCR

RFI line				
Gene symbol	VIP for	Low	High	*P* value
ACACB	FCR	0.92 + 0.09	0.68 + 0.08	**0.05**
AKAP12	RFI, FCRe	0.74 + 0.04	0.93 + 0.03	**0.008**
ATP1B1	FCR, FCRe	0.78 + 0.04	1.00 + 0.03	**< 0.001**
BLCAP	RFI, FCR	0.80 + 0.04	1.04 + 0.04	**< 0.001**
CD40	RFI	1.06 + 0.21	1.64 + 0.16	**0.03**
CSRNP3	RFI, FCR	1.33 + 0.20	2.49 + 0.17	**< 0.001**
EZR	RFI, FCR, FCRe	0.61 + 0.07	0.99 + 0.06	**< 0.001**
FKBP5	RFI, FCR, FCRe	0.74 + 0.14	1.48 + 0.12	**< 0.001**
FRAS1	RFI, FCR	1.06 + 0.10	1.17 + 0.09	0.40
FYN	FCRe, FCR	1.00 + 0.09	1.27 + 0.08	**0.02**
HSD11B1	FCR	1.29 + 0.29	2.81 + 0.25	**< 0.001**
IGF2	RFI, FCR	0.99 + 0.07	0.89 + 0.06	0.93
IL4R	RFI, FCR	0.94 + 0.08	1.22 + 0.06	**0.009**
MUM1	RFI	0.97 + 0.05	1.22 + 0.04	**0.001**
PDZD2	RFI, FCR	0.61 + 0.07	0.73 + 0.06	0.24
PHKB	RFI, FCR	0.74 + 0.04	0.87 + 0.03	**0.02**
PSEN1	RFI	0.79 + 0.05	0.96 + 0.04	**0.02**
RPL6	FCR, FCRe	0.90 + 0.03	0.91 + 0.03	0.90
SERINC3	RFI, FCR	0.61 + 0.05	0.87 + 0.04	**< 0.001**
SERPINA1	FCR, FCRe	1.32 + 0.14	0.89 + 0.14	**0.03**
SOCS6	RFI, FCR	0.48 + 0.06	0.58 + 0.05	0.17
TFG	FCR	1.04 + 0.04	1.18 + 0.04	**0.02**
TMED3	FCR	0.79 + 0.07	0.76 + 0.06	0.76
UGDH	RFI, FCR	0.87 + 0.08	1.14 + 0.07	**0.01**

*Abbreviations used*: *FCR* Feed conversion ratio, *FCRe* Net energy feed conversion ratio, *RFI* Residual feed intake, *VIP* Very important variable in prediction. Muscle transcriptomes from pigs (*n* = 71) of two lines divergently selected for RFI and reared under different conditions were considered. The qPCR technology was used to assess expression levels of target genes that were identified by a gradient tree boosting procedure as very important for prediction (VIP) of RFI, FCR or FCRe individual values. ANOVA was then used to evaluate the differences in expression levels of those genes between the two RFI lines

bold face highlights significant differences (*P* < 0.05) between lines

**Table 6:** Top contributing genes to the linear prediction of feed efficiency

RFI		FCR		FCRe	
24 VIP^1^	*R*^2^ = 0.73	*R*^2^ = 0.76		*R*^2^ = 0.75	
Subset^2^					
Gene	*P* value	Gene	*P* value	Gene	*P* value
FKBP5	< 0.001	FKBP5	< 0.001	FKBP5	< 0.001
SERINC3	0.02	MUM1	0.03	MUM1	0.04
IGF2	0.03	AKAP12	0.03	AKAP12	0.03
CSRNP3	0.03	FYN	0.03	PHKB	0.08
EZR	0.09	TMED3	0.08	SOCS6	0.07
RPL16	0.08	PHKB	0.08	FYN	0.08
		TFG	0.02	TFG	0.02
		SOCS6	0.07	TMED3	0.09
		ILR4	0.10	ILR4	0.10
		FRAS1	0.12	FRAS1	0.12
*R*^2^ = 0.58		*R*^2^ = 0.73		*R*^2^ = 0.71	

^1^A total of 24 target genes was used in a linear model for prediction of residual feed intake (RFI), feed-conversion ratio (FCR) and energy-based feed conversion ratio (FCRe)

^2^Stepwise selection was also used to retain the most significant variables in regression models for feed efficiency traits. Associated *P*-value for the entry of each variable (mRNA level of the gene) in the best model was indicated. All variables with *P* < 0.15 were considered

## Discussion

Because FE is recognized as a complex trait that involves many biological processes [3] and is influenced by genetics and environmental factors, FE related traits are difficult to predict. In this study, subsets of molecular predictors for different measures of FE were identified by using a machine learning method on muscle transcriptomes that were merged from original experiments to provide a larger number of animals and wider ranges of conditions. Very recently, Piles and colleagues [16] reported that machine learning algorithms provided good performance on RNAseq expression data to classify pigs into high or low RFI phenotypic groups when based on 100–200 very important genes expressed in liver (accuracy: 0.78) or duodenum (accuracy: 0.69). Although RNA sequencing data and microarray data are highly correlated, short and less abundant transcripts may have a higher possibility to be detected by the microarray approach [23]. Moreover, the *longissimus* muscle was found as the primary affected site, among four examined tissues including liver, by a divergent selection for RFI in growing pigs [4]. Therefore, we focused on microarray datasets obtained from *longissimus* muscle in different studies. Good accuracy was found (R^2^~0.65–0.70) for gradient tree boosting (GTB) models in prediction of RFI-BV, the measure of the net FE which was used as the selection criterion, and FCR, another measure of FE that is more easily obtained in most circumstances in pig farms. The error of prediction for FCR was less than 8% when calculated on all pigs. While data were included in the model without any (supervised) indication of genetic lines of origin, the deviation between observed and predicted values was higher for pigs from the high RFI line than for pigs from the low RFI line. Bad prediction concerned seven pigs with very high FCR, with no apparent bias arising from diets and feeding regimen. This suggests that the model cannot handle very high FCR values because they might be under a different metabolic control not captured in the dataset. In support, the metabolic phenotypes described in the referenced publications [21, 22] indicated higher glycaemia (1.7 vs 1.4 g/L) and leptinemia (1.9 vs 2.87 ng/L) but lower blood phospholipids concentrations (0.83 vs 1.0 g/L) in the seven pigs for which prediction of FCR largely deviated from the observations. These blood parameters may refer to something associated to energy homeostasis. Therefore, GTB models were built to predict FCR when expressed on a net energy basis. The prediction accuracy (R^2^) was slightly lower for FCRe than for FCR, and this may sign how the environment influences the two traits [24]. When a subset of the molecular predictors was further examined by qPCR to provide a technical validation of microarray data, linear regression models applied on mRNA levels of target genes confirmed the better accuracy of combination of several genes rather than one gene to predict RFI-BV, FCR and FCRe.

The molecular predictors identified as split variables for the different FE traits participated to a large variety of biological processes. Remarkably, most of these processes have been identified as pathways affected by FE divergence in pig muscle [4, 9, 25] and chicken breast [26]. Altogether, 56 predictors were common to RFI-BV and FCR, and six predictors were common between RFI-BV, FCR and FCRe. Finding common predictors of different FE traits is a challenge of interest, because the corresponding genes might be used as multiple biomarkers to reduce the effort of phenotyping in breeding programs. Among the six expressed genes proposed as common predictors for the three FE traits, FKBP5 (FKBP prolyl isomerase 5) is a member of the immunophilin protein family which plays a role in immune regulation and basic cellular processes involving protein folding and trafficking. In juvenile pigs, FKBP5 was proposed as a candidate gene for a better understanding of the stress response, notably due to its connexion with the glucocorticoid receptor [27]. Interestingly, it is known that FE is associated with the susceptibility to stress in cattle [28], and that RFI and cortisol response are also positively associated in rams [29]. Among the expressed genes identified as VIP which were further studied by qPCR, IL4R (interleukin 4 receptor), SERINC3 (serine incorporator 3) and SOCS6 (suppressor of cytokine signalling 6) also participated to immune and defense response. Difference in the activity of adaptive immunity has been previously underlined in pigs that were genetically [4] or phenotypically [9] different in FE. Another important gene predictor identified in this study was EZR (ezrin), which encodes a protein playing roles in cytoskeleton organization, cell adhesion, and morphogenesis. This gene was identified as a hub in a network of co-expressed genes involved in fat metabolism and highly related to RFI in cows [13]. In our study, different genes involved in the regulation of apoptosis/cell death were also proposed as important predictors of FE traits. In this category, CSRNP3 (cysteine and serine rich nuclear protein 3) codes for a transcriptional factor that was identified as lower expressed in muscle of low RFI pigs compared with high RFI pigs [8]. Importantly, IGF2, a member of the insulin-like growth factor family implicated in the regulation of cell development and muscle growth, was ranked among the top predictors of RFI-BV by GTB and linear regression models. The research on IGF2 gene polymorphism had revealed SNP with potential effects on growth rate and muscle mass in pigs [30, 31] and on FCR in beef cattle [32]. Moreover, RNA-seq analyses revealed an up-regulated expression of IGF2 in low RFI compared with high RFI pigs [10]. However, in this study, there was no significant difference in IGF2 expression level between low and high RFI pigs. Finally, FE predictors belonging to MAP kinase family, protein kinase and interleukins were found in muscle (this study) as in liver or duodenum [16].

Altogether, this study confirms that feed efficiency is underlined by variations in transcripts of different genes participating in many functional pathways. Because feed intake, BW gain, and body adiposity must be recorded for each animal to calculate feed efficiency during a test period, which is time-consuming, expensive and even difficult for group-reared animals, this study can be viewed as a proof of concepts that a small subset of expressed genes can be identified as a proxy for this complex trait. The loin muscle is largely affected by RFI selection [4]. It can be sampled at any stages of growth using biopsies, and more readily at market age during the slaughtering procedure, which can still have direct values to approximate the trait of interest. Therefore, it could be assumed that expressed muscle genes could serve to increase the accuracy of prediction of feed efficiency in next selection programs and (or) to indicate valuable biological pathways to update knowledge. Recent studies have rather proceeded using liver or digestive tract to identify genes associated with feed efficiency in growing pigs [16]. Finally, the GTB algorithm used for the prediction of feed efficiency traits is considered to be robust to partially inaccurate data and resistant to outliers in both predictors and target traits. In support, it gave a good fit of predicted to observed values even if the specific nature of the relationships between the predictor variables and the dependent variable is very complex. Mixing datasets encompassing different rearing conditions would have also maximized the chances for genericity of the candidates. However, the next steps will be to test the ability of the identified muscle genes to predict FCR in populations of pigs with different genetic structures.

## Conclusions

This study demonstrates the feasibility of finding few molecular predictors of complex traits such as feed efficiency from microarray datasets. Good accuracy of prediction models was obtained for RFI, FCR, and FCRe to a lesser extent, by using the expression levels of 6 to 267 expressed genes in *longissimus* muscle of pigs under different diets, feeding regimen and years. Other studies are required to validate these candidate genes in different studies and confirm the generality of the obtained predictions as planed via the combination of experimental designs. Our study can be viewed as a proof of concepts that small subset of expressed genes can be identified as a proxy of complex traits such as feed efficiency. Further studies must be conducted to apply the same procedures to peripheral blood as a relevant and easy sampling source of biological information.

## Methods

### Microarray data sets

Microarray data were available through GEO subseries accession numbers (http://www.ncbi.nlm.nih.gov/geo/query/acc.cgi?acc = GSE47769 for *n* = 23 pigs and GSE84092 for *n* = 48 pigs) repositories. Experiments used the Agilent-026440 *Sus scrofa* 44 K Oligo Microarray v2 and Agilent-037880/INRA *Sus scrofa* 60 K Oligo Microarray v1 (Agilent Technologies, Massy, France), respectively. The 037880/INRA microarray contained 60,306 porcine probes, and derived at 71% from the porcine commercial Agilent-026440 microarray (43,803 probes) with the remaining 29% corresponding to a set of probes enriched with immune system, muscle and adipose tissue genes. An updated (April 2016) annotation of the microarrays was used to check the correspondence between probe sequences and corresponding genes. On the 037880/INRA microarray (60 k), 63% of the probes are annotated, whereas on the Agilent-026440 microarray (44 k), 50% of the probes are annotated. The data were obtained from *longissimus* muscle from two purebred Large White pig lines divergently selected for RFI during 6 to 8 generations over three different trials. Across experiments, muscles were collected from barrows (*n* = 48) and females (*n* = 23) with body weight (BW) ranging from 80 to 115 kg. Pigs had free access to pelleted diets of standard composition (*n* = 39) or rich in dietary fibers and lipids (*n* = 24), and a subset of high RFI pigs (*n* = 8) were feed-restricted (− 10% of ad libitum intake) during the growing-finishing period.

In the merged dataset, pigs were classified into low or high RFI groups, respectively, according to their genetic lines (*n* = 40 for the high RFI line and *n* = 31 for the low RFI line). Selection principles have been described by Gilbert and colleagues [3]. Breeding values for RFI (RFI-BV) were newly estimated for these pigs by running a genetic evaluation of the trait combining data obtained on the full pedigree, based on their relative performance recorded in the selection farm (INRA GenESi, Rouillé, France). The RFI-BV were − 64.2 + 18.5 g/d on average in low RFI pigs and + 54.2 + 16.3 g/d in high RFI pigs. Feed conversion ratio (FCR) was calculated from individually measured daily feed intake (FI) and average daily gain (ADG) during the test periods as explained in the referenced publications [21, 22]. Net energy-based feed conversion ratio (FCRe) was obtained by considering net energy composition of the different diets together with daily FI and ADG of each pig.

### Descriptive statistics and gradient tree boosting procedure

#### Processing of molecular data

In the original publications, raw spot intensities obtained after hybridization reactions have been submitted to quality filtration (intensity, uniformity, saturation, and outlier detection) and intensities of filtered spots have been log2 transformed. Microarrays datasets from the different experiments were merged in a single new dataset by using the probes ID as references. This merged dataset included 20,405 annotated muscle probes expressed in the 71 pigs. In the merged dataset, the molecular data were normalized by mean centering, i.e. subtracting the mean value across all probes from all raw values for each pig sample, to obtain consolidated expression values across originally separated datasets. Master matrix of 71 pigs considered in the study with values of three feed efficiency traits, the metadata and the microarray expression profiles was provided in Additional file 6.

### Principal component analysis (PCA)

The variables were represented on a same frame to describe the heterogeneity and consistency of the data, and to define the nature and importance of the links between the variables. Principal component analysis (PCA) was performed as an unsupervised method that summarizes the large number of expressed probes into a set of uncorrelated principal components (PC) by means of their covariance structure. No outliers were identified. Pigs’ coordinates for each PC were used to identify which PC separated pigs according to RFI groups. Pearson correlation coefficients were also calculated between FCR, FCRe and PC.

### Gradient tree boosting

The Gradient Tree Boosting (GTB) procedure was used to identify very important variables in prediction (VIP) for traits of interest (RFI, FCR and FCRe). The GTB is an advanced machine-learning algorithm for regression analysis that offers a more powerful data mining tool to generate accurate models when compared with single models or by ensembles such as bagging or conventional boosting [33–35]. The algorithm typically generates thousands of small decision trees built in a sequential error–correcting process to converge to an accurate model [33]. The model is similar to Fourier or Taylor series, which is a sum of factors that becomes progressively more accurate as the expansion continues. In other terms, the GTB machine builds a sequential series of decision trees, where each tree corrects the residuals in the predictions made by the previous trees; after each step of boosting, the algorithm scales the newly added weights, which balances the influence of each tree. Therefore, at each stage of gradient boosting, it was assumed that there was some imperfect model so that the gradient boosting algorithm was improving it by constructing a new model that added an estimator to provide a better model. Moreover, the accuracy of the algorithm was typically improved by introducing randomization through training the base learner on different randomly selected datasets at each iteration. As opposed to neural networks, this methodology is not sensitive to data errors and needs no time-consuming data preparation, pre-processing or imputation of missing values. In the present study, GTB prediction models were generated with the Salford Predictive Modeler 8.0 (SPM 8.0®) software. In the current study, about 1114 to 1500 trees were created for each FE trait, with each tree typically containing about six terminal nodes as recommended for boosting [35]. Each tree was built on a randomly selected bootstrap sample, by using 70% of the original dataset for learning (*n* = 50 pigs) and a randomly selected subset of variables. Consequently, each bootstrap sample called “out-of-bag” data (OOB) excluded 30% of the data that were used for testing in the validation step (*n* = 21 pigs). The random partition of the muscle samples between learning and validation sets in relation to the datasets of origin was carefully checked. Significant variables were selected using the Gini index to evaluate discriminative ability defined as:

$$ \mathrm{Gi}=1\hbox{-} \sum j\kern0.5em p\mathrm{j}\kern0.5em \left(j|t\right) $$

Where pj (푗 | 푡) is the estimated class probability for feature 푡 or node 푡 in a decision tree and 푗 is an output data. Only the variables that improved Gini index and minimized the OOB error rate were retained as VIP. The root mean square error (RMSE) was then calculated as the square root of the difference between the realized and the predicted observation within the OOB data after permuting each predictor variable in the training dataset divided by the number of trees. The adjusted coefficient of determination (R^2^) was also computed.

For FCR, predicted (X) and observed (Y) values were further compared (X - Y), using the GLM procedure. The model was considered unbiased when the intercept was not different from 0 and the slope was not significantly different from 1. The quality of the relationships was evaluated on the basis of RMSE of prediction (RMSEP), obtained by a leave-one-out cross-validation from the value of the predicted residual sum of squares (PRESS) statistics [36]. Model evaluation was performed for all pigs, and RFI line by RFI line.

### Functional pathways represented across the VIP

The gene ontology terms for biological processes (GOBP) were automatically searched within each list of VIP which were uploaded by their official gene symbol. The Database for Annotation, Visualization and Integrated Discovery (DAVID) bioinformatics resource database (v6.7; http://david.abcc.ncifcrf.gov) was used, with *Homo sapiens* as background for mapping and enrichment analysis. The results were downloaded using the “Functional annotation clustering” option of the DAVID tool, and medium clustering stringency was selected to generate the functional groups across the genes based on a priori knowledge [37]. For each term, the enrichment (E) score (measured by the geometric mean of the EASE score of all enriched annotations terms for each cluster) and the modified Fisher exact *P*-value were obtained. Altogether, E > 1.0 and *P* < 0.05 were considered to list the significantly top-enriched clusters of genes.

### Commonalities across VIP between feed efficiency traits

To deduce the commonalities across the VIP retained for the different FE traits (RFI, FCR and FCRe), the free online VENNY tool [http://bioinfogp.cnb.csic.es/tools/venny/index.html] was used to handle each VIP by its corresponding gene name, and then, to edit VENN diagrams.

### Quantitative real-time PCR (qPCR)

Expression levels of 24 target genes identified as common VIP for at least two FE traits and (or) participating to pathways judged as biologically-relevant for one FE trait, were further determined in the same *longissimus* muscle samples (*n* = 71) where microarrays data have been generated. The SmartChip Real-Time PCR system (Wafergen) available at the Human and Environmental Genomics (GEH) technological core facilities (Rennes, France), was used. Total RNA was extracted as described previously [4, 8]. First-strand cDNA synthesis was performed with 1 μg of total RNA used for microarray analysis, by using High Capacity RNA to cDNA Kit (Applied Biosystems, Foster City, USA). Primers (Additional file 7) were designed from porcine sequences available in Ensembl or NCBI databases using Primer Express® v3.0 software (Applied Biosystems). Detailed information on the primer sequences (forward and reverse) is provided in Additional file 4. Amplification reactions were carried out using LightCycler 480 SYBR Green 1 Master (Roche Diagnostics, Meylan, France) with a final cDNA concentration of 1 ng/μL and a primer concentration of 500 nM dispensed using the WaferGene SmartChip Multisample Nanodispenser. Amplification conditions were as follows: 5 min at 95 °C followed by 50 cycles of 30 s at 95 °C, 30 s at 60 °C and 30 s at 72 °C, followed by 15 s at 95 °C and 1 min at 60 °C. Specificity of the amplification products was checked by dissociation curve analysis. As stated by the GeNorm algorithm (https://genorm.cmgg.be/), RPL4 and TBP1 were identified as the most stable housekeeping genes among other tested reference genes, and were used to calculate the normalization factor (NF). For each gene, the normalized expression level N was calculated according to the formula: N = E-ΔCq (sample-calibrator)/NF where E was calculated from the slope of calibration curve, Cq was the quantification cycle, and the calibrator was a newly generated biological sample constituted by the pool of the 71 samples. For all studied genes, E was between 1.82 and 2.10.

### Linear regression models

Differences in expression values of the 24 genes between low and high RFI groups were first evaluated using variance analysis (GLM procedure) for the effect of RFI line on the SAS software (SAS, Cary NC). Then, regression models on mRNA levels of these 24 genes were applied to determine the prediction accuracy for RFI, FCR and FCRe, respectively, considering RSQUARE selection (i.e., the independent variables that best predict the dependent variable by linear regression) or stepwise option (i.e., the variables are added one by one to the model). As recommended, *P* < 0.15 was used as threshold to retain significant variables in the stepwise regression model. Additional regression analyses were also performed using final BW as an explicative factor of FCR in supplement to expression levels of the target genes.

## Additional files

Additional file 1 Plot of the first two principal components unraveling whole variability in the merged molecular dataset (DOCX 58 kb)

Additional file 2 Top-ranked genes contributing to RFI prediction (XLSX 36 kb)

Additional file 3 Top-ranked genes contributing to FCR prediction (XLSX 38 kb)

Additional file 4 Top-ranked genes contributing to FCRe prediction (XLSX 33 kb)

Additional file 5 Regression slopes between observed and predicted values of feed conversion ratio (FCR) for pigs categorized according to their genetic lines and dataset of origin. (PDF 287 kb)

Additional file 6 Master matrix with values of three feed efficiency traits, the metadata and the microarray expression profiles. (XLSX 14964 kb)

Additional file 7 Primers for studying target genes by qPCR (DOCX 19 kb)

## Data Availability

The datasets analyzed in this study are available in the GEO subseries accession numbers (http://www.ncbi.nlm.nih.gov/geo/query/acc.cgi?acc = GSE47769 and GSE84092) repositories. All data generated during this study are included in this published article and its supplementary information files.

## Abbreviations

ADG: Average daily gain
BW: Body weight
DAVID: Database for annotation, visualization and integrated discovery
FCR: Feed conversion ratio
FCRe: Net energy-based feed conversion ratio
GTB: Gradient Tree Boosting
OOB: out-of-bag
PC: principal component
PCA: Principal component analysis
qPCR: quantitative real-time polymerase chain reaction
RFI: Residual feed intake
RMSE(P): Root mean square error (of prediction)
VIP: Very important variable in prediction
