# Supplementary material for: Investigation of muscle transcriptomes using gradient boosting machine learning identifies molecular predictors of feed efficiency in growing pigs
Source: BMC Genomics. 2019 Aug 17;20:659. doi: 10.1186/s12864-019-6010-9 (PMC6697907; doi:10.1186/s12864-019-6010-9)
Supplement: Supplementary file 1 — Plot of the first two principal components unraveling whole variability in the merged molecular dataset (DOCX 58 kb) [file 12864_2019_6010_MOESM1_ESM.docx]

Suppl. File 1

Legend: Plot of the first two principal components unraveling whole variability in the merged molecular dataset

Pigs are represented on the scatter plot created with the first two principal components (PC) of a Principal Component analysis (PCA) which aggregated the whole transcriptomic data (20,405 annotated expressed probes) in the longissimus muscle of different studies. Full description of the experimental designs (including generation of selection, sex, diet composition, feeding regimen, etc.) can be found in the referenced publications (dataset 1: Vincent et al., 2015; dataset 2: Gondret et al., 2014; 2016).

Labels: green dot: low RFI pigs – dataset 1 (label PF_N° pig); purple dot: high RFI pigs – dataset 1 (label PF_N° pig); blue dot: low RFI pigs – dataset 2 (label FI_N° pig) ; red dot: high RFI pigs – dataset 2 (label PF_N° pig).
