# Supplementary material for: Investigation of muscle transcriptomes using gradient boosting machine learning identifies molecular predictors of feed efficiency in growing pigs
Source: BMC Genomics. 2019 Aug 17;20:659. doi: 10.1186/s12864-019-6010-9 (PMC6697907; doi:10.1186/s12864-019-6010-9)
Supplement: Supplementary file 5 — Regression slopes between observed and predicted values of feed conversion ratio (FCR) for pigs categorized according to their genetic lines and dataset of origin. (PDF 287 kb) [file 12864_2019_6010_MOESM5_ESM.pdf]

Suppl. File 5

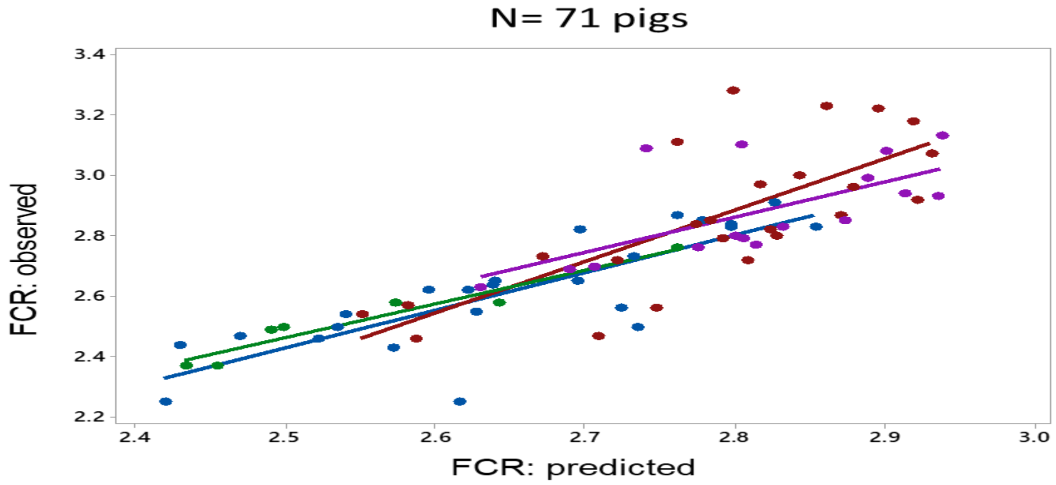

Legend: Plot of observed and predicted FCR (feed conversion ratio) values, with pigs labelled according to their genetic line (RFI, residual feed intake) and experiments (datasets 1 or 2). Slopes are indicated, together with coefficients of regression (into brackets). $R^2 = 0.70$ when evaluated in the 71 pigs. Labels : green dot: low RFI pigs – dataset 1 ($R^2 = 0.93$); purple dot: high RFI pigs – dataset 1 ($R^2 = 0.43$); blue dot: low RFI pigs – dataset 2 ($R^2 = 0.67$) ; red dot: high RFI pigs – dataset 2 ($R^2 = 0.53$).

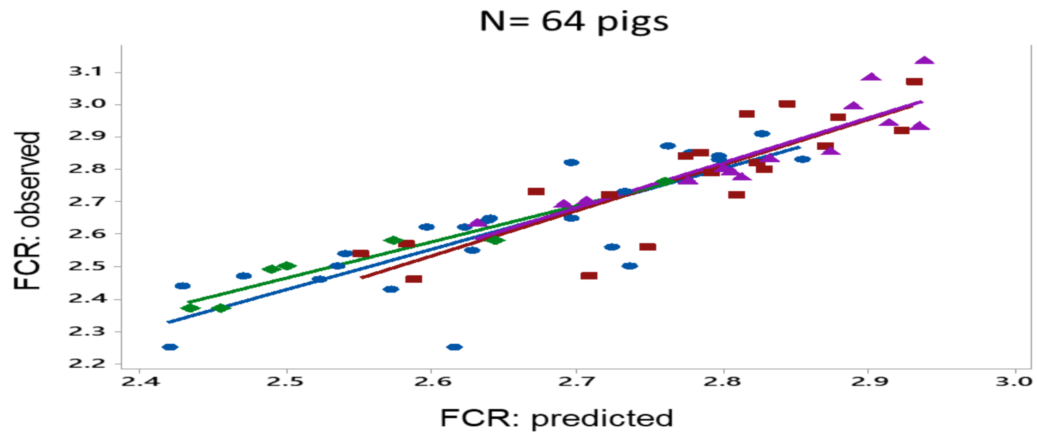

Legend: Plot of observed and predicted FCR (feed conversion ratio) values, with pigs labelled according to their genetic line (RFI, residual feed intake) and experiments (datasets 1 or 2). Slopes are indicated, together with coefficients of regression (into brackets). $R^2 = 0.80$ when evaluated in the 64 pigs. Labels: green diamond: low RFI pigs – dataset 1 ($R^2 = 0.92$); purple triangle : high RFI pigs – dataset 1 ($R^2 = 0.81$); blue dot : low RFI pigs – dataset 2 ($R^2 = 0.67$); red square: high RFI pigs – dataset 2 ($R^2 = 0.72$).

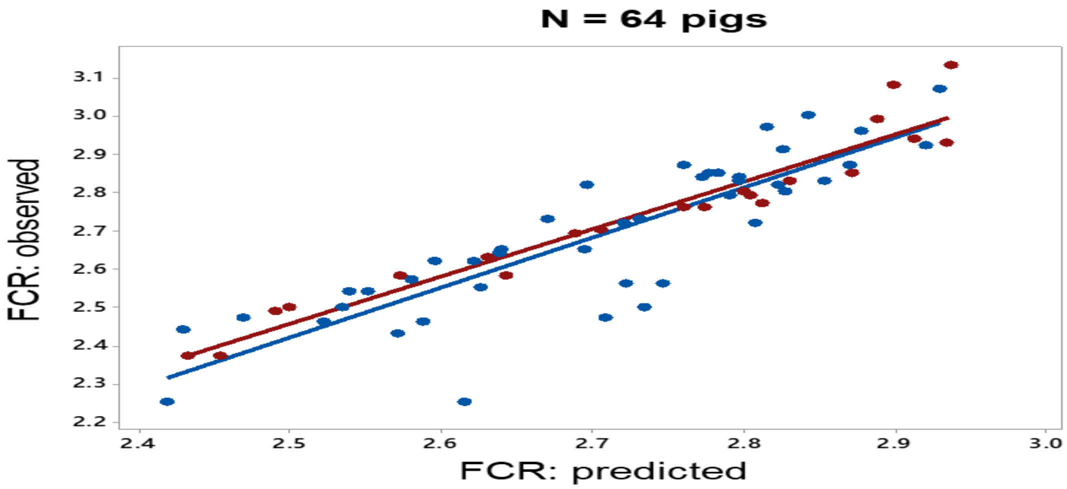

Legend: Plot of observed and predicted FCR (feed conversion ratio) values, with pigs labelled according to dataset of origin. Slopes are indicated together with coefficients of regression (into brackets). $R^2 = 0.80$ when evaluated in the 64 pigs. Labels: red dot: dataset 1 ($R^2 = 0.93$) – blue dot: dataset 2 ($R^2 = 0.73$).
