# Supplementary material for: Investigation of muscle transcriptomes using gradient boosting machine learning identifies molecular predictors of feed efficiency in growing pigs
Source: BMC Genomics. 2019 Aug 17;20:659. doi: 10.1186/s12864-019-6010-9 (PMC6697907; doi:10.1186/s12864-019-6010-9)
Supplement: Supplementary file 7 — Primers for studying target genes by qPCR (DOCX 19 kb) [file 12864_2019_6010_MOESM7_ESM.docx]

**Additional file 7**: Primers for studying target genes by qPCR

| Gene symbol | | Gene name | | Accession number^1^ | | Primer and probe sequences | |
| --- | --- | --- | --- | --- | --- | --- | --- |
| ACACB | | Acetyl-CoA carboxylase beta | | CV869475 | | F^2^: CCATCCGAGAGAACATCAAATACTT  R^3^: TGAACCAGGCCTCTGATGGT | |
| AKAP12 | | A-kinase anchoring protein 12 | | XM_001927265.7 | | F: GAGCCCAAGCGCAAGGTT  R: GATGACCCCACACAAATTAAAGC | |
| ATP1B1 | | ATPase, Na^+^/K^+^ transpor-ting, beta 1 polypeptide | | NM_001001542 | | F: GGCCCCACCAGGATTAACAC  R: GCTTTGGGGATCATTAGGACGA | |
| BLCAP | | bladder cancer associated protein | | XM_001928462.4 | | F: GGCGTTGTGGGCACCTAA  R: CCGTCTTCTGCTTCCTTGGA | |
| CD40 | | TNF receptor superfamily member 5 | | NM_214194.1 | | F: GCGCCTGTGAAAGTTGCA  R: CTCTGTCGCCATCTGCTTGA | |
| CSRNP3 | | cysteine-serine-rich nuclear protein 3 | | ENSSSCT00000017319 | | F: TCAATCCATCCACTTCCAATCA  R:GCCGCTTCTCCCTTTTGAG | |
| EZR | | Ezrin | | XM_013992459.2 | | F: GCCGAAACCAATCAACGTCC  R:TCCTGTTGTGTTTGGCTGGA | |
| FKBP5 | | FK506 Binding protein 5 | | NM_001315611.1 | | F: GTCCACAGCAGCATCACTTG  R:GTTCGGGAGGTGGCTAAGTC | |
| FRAS1 | | Fraser extracellular matrix complex subunit 1 | | XM_013978814.2 | | F:AGCACAACCGTCTGGACAATC  R: CCCTCGTCAACCGTGATGA | |
| FYN | | proto-oncogene, Src family tyrosine kinase | | NM_001080206.2 | | F: GCCAAGGACTCACCGTCTTC  R:TCACTCCTGTTCCTCCTCTCGTA | |
| HSD11B1 | | hydroxysteroid 11-beta dehydrogenase 1 | | NM_214248.3 | | F: CATAGACACAGACACGGCCA  R: CAGGGCGCATTCTTCCTTTG | |
| IGF2 | | Insulin-like growth factor 2 | | NM_213883.2 | | F: CGTGCTGCTATGCTGCTTAC  R: CCGGCCTGCTGAAGTAGAAG | |
| IL4R | | interleukin 4 receptor | | NM_214340.1 | | F:CCCCACGGATTTCAGAATCTATA  R:ACGCGTGCGCTGTAAGC | |
| MUM1 | | melanoma associated antigen (mutated) 1 | | XM_021084240.1 | | F: ACCACTTGCCACATGAGTCT  R:CAGTTCCGGCCAAGACCTTT | |
| PDZD2 | | PDZ domain containing 2 | | XM_021077090.1 | | F: CCTCCTGGCATCTACATTCACA  R:GCGGACGTTGACAGAGTTCA | |
| PHKB | | phosphorylase kinase regulatory subunit beta | | XM_021094235.1 | | F: TGGCAACCAGGGCTGTTC  R: TCTGCCGGTTGCGATTGT | |
| PSEN1 | | presenilin 1 | | XM_005666340.1 | | F:GTCACGATCTGCTGTACAGGATCT  R: ATCCAAGTTTTACTCCCCTTTCTTC | |
| RPL6 | | ribosomal protein L6 | | NM_001044542.1 | | F: ACAGAGGCAAGAGGGTCATTTTC  R:CAGAGGTCCAGTCACAAGTAACAAG | |
| SERINC3 | | serine incorporator 3 | | XM_021077977.1 | | F: TCCGGGAGATTCTCGGTAGT  R: CACAAAGGCATGGAACCCAG | |
| SERPINA1 | | serpin family A member 1 | | NM_214395.2 | | F: TGAGCTTGACAAAGACACCGTATT  R: GCTTCTCCCATTTGCCTTTAAA | |
| SOCS6 | | suppressor of cytokine signaling 6 | | HM135392.1 | | F: CGCTGCCGGAAAATGGC  R: CTCAGACATCTGGGGAGGCT | |
| TFG | | TRK-fused gene | | XM_021070561.1 | | F: TTCTTCCTCCTCTGCAGCTC  R:CATCTGACCTTCACTCTGACCT | |
| TMED3 | | transmembrane p24 trafficking protein 3 | | NM_001204363.1 | | F:ACGAGCCTCCTATTCTCCCA  R:GACTCCATCTGGGTGAGAGC | |
| UGDH | | UDP-glucose 6-dehydrogenase | | XM_003356899.5 | | F: TGGTGCTTGGAAGTGGTACA  R: GTGGGTCCTCCAACATAGCC | |
| RPL4^4^ | | Ribosomal Protein L4 | | ENSSSCG00000004945 | | F:AGGAGGCTGTTCTGCTTCTG  R: TCCAGGGATGTTTCTGAAGG | |
| TBP1^4^ | | TATA box binding protein | | DN110073 | | F: AACAGTTCAGTAGTTATGAGCCAGA  R: AGATGTTCTCAAACGCTTCG | |

^1^Accession number in the National Center for Biotechnology Information database (<http://www.ncbi.nlm.nih.gov/gene>) or Ensembl project database (<http://www.ensembl.org/Sus_scrofa/Info/Index>) for pig sequences

^2^F = forward primer; ^3^R = reverse primer; ^4^Genes used as references for qPCR normalization.
